# Why might fears and worries persist after a pain education–grounded multimodal intervention for chronic back pain? A qualitative study

**DOI:** 10.1097/PR9.0000000000001197

**Published:** 2024-11-13

**Authors:** Rodrigo R. N. Rizzo, Benedict M. Wand, Hayley B. Leake, Edel T. O'Hagan, Adrian C. Traeger, Sylvia M. Gustin, G. Lorimer Moseley, Saurab Sharma, Aidan G. Cashin, Matthew K. Bagg, James H. McAuley, Samantha Bunzli

**Affiliations:** a School of Health Sciences, Faculty of Medicine, University of New South Wales, Sydney, Australia; b Centre for Pain IMPACT, Neuroscience Research Australia, Sydney, Australia; c Faculty of Medicine, Nursing & Midwifery and Health Sciences, The University of Notre Dame Australia, Fremantle, Australia; d IIMPACT in Health, University of South Australia, Kaurna Country, Adelaide, Australia; e Pain Education Team to Advance Learning (PETAL) Collaboration; f Westmead Applied Research Centre, Faculty of Medicine and Health, The University of Sydney, Sydney, Australia; g School of Public Health, Faculty of Medicine and Health, The University of Sydney, Sydney, Australia; h NeuroRecovery Research Hub, School of Psychology, University of New South Wales, Sydney, Australia; i School of Health Sciences and Social Work, Griffith University, Nathan Campus, Queensland, Australia; j Physiotherapy Department, Royal Brisbane and Women's Hospital, Queensland, Australia

**Keywords:** Patient education, Qualitative research, Low back pain, Chronic pain, Pain management

## Abstract

**Introduction:**

The effect of pain education (PE) on pain intensity and function diminishes after a few months in people with chronic low back pain (CLBP). One possible explanation is the return of underlying fears and worries related to the condition.

**Objective:**

To explore topics related to participants' beliefs and feelings that might explain why fears and worries persist after a PE-grounded intervention for CLBP.

**Methods:**

We conducted a qualitative study involving semistructured interviews with participants from the active arm of a randomised controlled trial who received an individualised PE-grounded intervention for adults with CLBP. We used reflexive thematic analysis with an inductive approach.

**Results:**

Twenty participants were interviewed (9 women and 11 men, median age = 54 years, median pain duration = 4 years, 13 reporting at least 30% pain reduction on the trial primary outcome). Three themes were identified: 1) “Are you implying my pain is not real?”: a few participants believed the validity of their pain was being questioned. 2) “You don't understand, my pain is different”: most participants considered the influence of an altered nervous system but did not exclude the possibility of having structural and biomechanical influences for the persistence of their back pain. 3) “I am unsure how to fit it into my daily life”: fear and worries persisted when participants could not figure out how to apply an alternative way of making sense of pain in their daily lives.

**Conclusion:**

Patients' perceptions about PE should be monitored and might be addressed with communication strategies, educational content that matches patients' characteristics, and reinforcements over time.

## Abstract 2

Supplemental Digital Content is Available in the Text.

Fears and worries may persist after pain education when pain experiences are invalidated, explanations and experiences are mismatched, or not incorporated into daily life.

## 1. Introduction

Clinical practice guidelines for nonspecific chronic low back pain (CLBP) encourage effective, safe, and cost-effective nonpharmacological interventions such as patient education.^15^ Patient education is a flexible intervention that can be applied in different clinical settings, delivered by various health professionals and is best used in combination with other interventions.^13^ Patient education has markedly evolved over the last 2 decades. Historically, patient education for CLBP focussed on explaining the structure and function of the spine (biomechanical patient education). In this type of education, patients are informed about the postures and movements supposed to protect the back from (further) damage^14^ Consistent with evidence indicating that low back pain is a multidimensional experience,^36^ contemporary “biopsychosocial” patient education seeks to address fears and worries about the condition, explain that the social context influences pain, and encourage patients to return to normal levels of activity and social participation (biopsychosocial patient education).^26^

Pain education (PE)^8^ is one type of biopsychosocial patient education. It seeks to shift how people conceptualise their pain away from a marker of tissue damage or disease towards a marker of the perceived need to protect the body by expanding people's understanding of the biological processes underpinning pain and the potential impact on pain of beliefs, emotions, behaviours, and lifestyle choices.^20^ Pain education imparts greater benefits for a range of outcomes, including fear and worries, than biomedical patient education.^22^ Although there is evidence that societal beliefs about back pain may have changed over time,^27^ many people still hold strong biomedical views about back pain and uncertainties about its management.^12,27^ This lack of alignment between PE and the dominant sociocultural view may interact with PE's longer-term effects in clinical practice.^21^ Systematic reviews show that PE combined with other interventions improves pain and disability compared with a range of control groups without PE at 3 months in people with CLBP.^38^ However, its effectiveness may diminish after this 3-month period.^38^ The observation that short-term improvements in pain intensity and disability through PE can be partly explained by reductions in fears and worries about CLBP^10^ raises the possibility that diminished long-term effects reflect the return or persistence of underlying fears and worries related to CLBP. A high-quality randomized controlled trial showed that a PE-grounded intervention could improve pain and function by reducing fear and worries in people with CLBP.^1,10^ Participant perceptions of what was missing from the treatment and how PE-grounded intervention compares to other treatments' were previously reported in our study.^29^ This study was conducted through a different lens to explore the challenges people face with PE-grounded intervention associated with fears and worries during and after the intervention.

## 2. Material and methods

### 2.1. Study design

This is a qualitative study using semistructured interviews. The master protocol was prospectively registered on the Open Science Framework (https://bit.ly/3GeuuY1). This study was conducted following the Declaration of Helsinki and approved by the Research Ethics Committee of the University of New South Wales (HC200119). The reporting complies with the Standards for Reporting Qualitative Research (SRQR).^23^

### 2.2. Research team and assumptions

The multidisciplinary research team involved physiotherapists (R.R.N.R., B.M.W., H.B.L., E.O.H., G.L.M., A.C.T., S.B., S.S., M.K.B.), psychologists (J.M. and S.M.G.), and an exercise physiologist (A.G.C.). The team has research interests in studying treatments for back pain, the biology of pain, pain education, and/or experience in qualitative research approaches. The interviewer and primary analyst (R.R.N.R.) received formal training in qualitative research and support from the last author (S.B.), who has extensive experience in qualitative research. R.R.N.R. is a male clinician with over 15 years of experience using psychologically informed interventions to manage people with CLBP and was a PhD candidate when conducting the interviews and analyses. This study was underpinned by a relativist ontological perspective, which assumes that reality depends on context,^5^ and an interpretivist epistemological position, which assumes that the natural interaction between the researcher and participant shapes knowledge to inform clinical practice.^35^

### 2.3. Participants and recruitment

Participants were identified from their previous participation in a single-centre randomised controlled trial of graded sensorimotor retraining—incorporating an individualised PE component—compared with a sham intervention (RESOLVE trial) conducted in Sydney, Australia, from 2015 to 2019.^16^ Participants were people with nonspecific CLBP (pain persisting for longer than 3 months without identified specific spinal pathology).^16^ Following the end of the trial, participants allocated to graded sensorimotor retraining (the active arm of the trial) were invited to participate in this qualitative study.

Purposive sampling was applied to recruit a diverse sample in terms of age, sex, and baseline pain characteristics. Participants were balanced according to changes from baseline for pain intensity at the 18-week follow-up, which was the time point of the primary outcome of the trial (ie, at least 30% pain reduction or less than 30% pain reduction on the 0–10 Pain Numerical Rating Scale [NRS]). A previous mediation analysis study with the trial population showed that participants who did not improve in fears and worries outcomes were less likely to report reduction in pain intensity and disability.^10^ For this reason, we assumed that the sample (with different levels of pain intensity) would include people whose fear and worries improved and did not improve with the PE-grounded intervention. A research assistant invited trial participants through text messages to participate, sent responders consent forms, and scheduled all interviews. The recruitment was conducted after the 52-week trial follow-up by a research assistant not involved in the interviews to maintain blinding in the randomised controlled trial. Participants included in the current qualitative study were the same as in a complementary qualitative study that investigated the acceptability of PE-grounded multimodal intervention.^29^ We used the same data set but formulated a different research question and conducted a separate analysis following best practices of conducting qualitative analysis using the same data set.^30^

### 2.4. Pain education–grounded multimodal intervention

Participants attended 50–60-minute individualised sessions weekly for 12 weeks in the PE-grounded multimodal intervention—the first 2 sessions involved solely PE.^19^ In sessions 3 to 12, PE was integrated and reinforced with other intervention components, including premovement approaches (ie, sensory precision training and mental rehearsal of movement) and graded and individualised movement and loading training that progress toward patient-valued functional goals.^1^ Clinicians used a flexible treatment manual and multimedia educational materials to deliver PE.^8,18,19^ Homework was assigned in each session, which included reading sections from Butler and Moseley's Explain Pain book^7^ and watching selected freely available videos related to PE from YouTube. Multiple trained physiotherapists and one exercise physiologist delivered the intervention. Clinicians were trained to use active listening, validate, reassure, and individualise the intervention to participants' needs. The full description of the PE is presented elsewhere.^1^

### 2.5. Interview and data collection

A semistructured interview guide was developed to explore participants' experiences with the PE-grounded multimodal intervention (Supplement material 1, http://links.lww.com/PR9/A253). Interviews adopted a flexible conversation style to facilitate the emergence of topics related to participants' experiences, challenges, and points of view about the PE-grounded multimodal intervention.^5^ This study used the same interview guide and dataset from a previous qualitative study that investigated the acceptability of the PE-grounded intervention using a theoretical framework.^29^ The interview guide was piloted in the first 3 participants (with no changes; therefore, it was included in the final sample). Author R.R.N.R. conducted all interviews through telephone in 2020. R.R.N.R. did not have any preexisting relationship with the participants and was blind to participants' clinical characteristics.

All participants provided verbal informed consent for the qualitative study immediately before the interview. Interviews were audio-recorded using a voice recording device, transcribed using Microsoft Office Word, and entered into NVivo 10 to facilitate the analyses.

### 2.6. Data analysis

Data collection and analysis were interactive and conducted in parallel. Data were analysed using Reflexive Thematic Analysis with an inductive approach described in Box 1.^3,4,6,34^

Box 1. **Inductive approach process for data analysis**

(1) *Familiarisation:* R.R.N.R. familiarised themselves with the data by listening to the interviews and reading the transcripts.
(2) *Coding with a broad question in mind*: R.R.N.R. performed a systematic coding process that started widely to explore the overarching question: “What are people's beliefs and feelings about back pain 12 months after participating in an intensive PE-grounded multimodal intervention?.”
(3) *Coding with a narrow question in mind:* R.R.N.R. conducted more narrow coding guided by the question: “Why might fears and worries persist after a PE-grounded multimodal intervention?” to identify implicit meanings that went beyond the explicit content of the data.
(4) *Preliminary themes:* codes were collapsed into categories and patterns (preliminary themes) through consensus discussion between R.R.N.R., S.B., and H.B.L. Exceptions to the patterns were explicitly sought to define overarching themes and identify minor themes.
(5) *Themes*: R.R.N.R. continued the process and presented the collapsed codes to members of the wider research team, whose contrasting views explicitly sought to challenge interpretations and ensure that the quotes were consistent with the codes and themes.
(6) *Final themes:* R.R.N.R. reviewed, named, and described the final themes. Saturation was achieved when authors acknowledged the attainment of sufficient conceptual depth to generate meaningful insight to inform care and clinical practice.

## 3. Results

Characteristics of the included participants are described in Table 1. Thirty potential participants were contacted through text messages. Twenty responded and agreed to participate in the study. The sample was 9 women (45%) and 11 men (55%), with a median (range) age of 54 (35–70) years and a median (range) duration of back pain of 4 (0.5–23.0) years. Thirteen participants (65%) reported at least 30% pain reduction on the 0 to 10 NRS at the 18-week follow-up. Interviews took place between 1.1 and 2.5 years after the participants' first session of the 12-week program. The median (range) duration of the interviews was 47 (37–77) minutes.

**Table 1:** Participant characteristics.

Participant	Sex	Age (y)	Work status (during the education programme)	Duration of LBP (y)	Pain intensity (change from baseline to 18 wk post programme on the 0–10 PNRS)	Disability (change from baseline to 18 wk post programme, 0–24 RMDQ)	Global Back Recovery scale (at 18 wk after the programme; on the −5 to 5 GBR scale)	Pain knowledge (at 18 wk after the programme; 0–18 scale)	Fear (at 18 wk after the programme; on the 17–68 TSK)	Worries (at 18 wk after the programme; on the 0–52 PCS)	Time elapsed since concluding PE-grounded intervention (years after the programme)
P01	M	35	Casual employed	12	**−** **6 (−** **100%)**	**−4 (−80%)**	**5**	13	26	3	1.1
P02	F	56	Part-time employed	3	**−7 (−87%)**	**−8 (−100%)**	**4**	12	26	2	2.0
P03	M	70	Not in paid employed	5	**−2 (−40%)**	**−8 (−66%)**	3	11	30	4	1.6
P04	F	50	Not in paid employed	1	**−6 (−85%)**	**−11 (−100%)**	**4**	15	25	4	1.2
P05	F	49	Full-time employed	4	**−3 (−75%)**	**−2 (−100%)**	**4**	15	20	1	1.6
P06	F	45	Part-time employed	23	0 (0%)	**−9 (−75%)**	3	12	23	13	1.3
P07	F	40	Part-time employed	23	+1	**−12 (−85%)**	2	12	33	13	1.3
P08	M	57	Full-time employed	4	0 (0%)	**−4 (−100%)**	2	12	27	0	2.2
P09	F	46	Full-time employed	20	+2	+4	**5**	14	21	1	2.3
P10	M	43	Full-time employed	13	**−5 (−55%)**	**−16 (−84%)**	3	11	26	6	2.3
P11	M	53	Full-time employed	3	0 (0%)	**−9 (−100%)**	3	16	20	2	2.2
P12	M	69	Not in paid employed	2	**−5 (−71%)**	**−10 (−100%)**	**4**	13	31	7	2.0
P13	M	60	Part-time employed	10	+3	+1	3	11	33	10	2.3
P14	F	62	Not in paid employed	0.9	**−4 (−80%)**	**−9 (−69%)**	**4**	10	23	18	2.3
P15	M	58	Full-time employed	1	**−3 (−42%)**	**−7 (−36%)**	2	10	27	5	1.1
P16	F	64	Not in paid employed	0.5	**−1 (−33%)**	**−9 (−81%)**	1	14	32	5	2.2
P17	F	55	Part-time employed	3	−1 (−20%)	**−6 (−50%)**	0	12	32	4	1.2
P18	M	67	Not in paid employed	4	**−5 (−62%)**	**−16 (−94%)**	3	13	26	1	2.5
P19	M	42	Full-time employed	4	**−1 (−25%)**	**−11 (−75%)**	2	16	26	0	1.2
P20	M	51	Full-time employed	3	**−1 (−33%)**	**−6 (−100%)**	3	5	36	5	2.0

Bold values mean clinically important change for pain intensity (at least 30% of improvement on the 0–10 NRS), disability (at least 30% of improvement on the 0–24 RMDQ), or Global Back Recovery (>3 on the −5 to 5 GPE scale which varies from vastly worse to completely recovered).

GBR, Global Back Recovery scale, positive numbers mean better results for recovery; PE, pain education; pain knowledge, Neurophysiology Pain Questionnaire, greater numbers mean greater pain knowledge; PCS, Pain Catastrophizing Scale, higher scores are worse; PNRS, Pain Numerical Rating Scale, negative numbers mean better results for pain intensity; RMDQ, Roland Morris Disability Questionnaire, negative numbers mean better results for disability; TSK, Tampa Scale of Kinesiophobia, higher scores are worse.

We identified 3 themes: (1) “Are you implying my pain is not real?” when participants demonstrated concerns that the intervention questioned the validity of their pain experience. (2) “You don't understand; my pain is different” when participants reported fear or worries about the structural influences for the persistence of their back pain. (3) “I am unsure how to fit it into my daily life” when participants expressed fear and worries of future pain because they could not apply the alternative way of making sense of pain in their daily lives. We described some quotes narratively in the Results section and provided additional quotes in Supplement 2, http://links.lww.com/PR9/A253.

### 3.1. Are you implying my pain is not real?

A trusting relationship with the clinician delivering the PE-grounded intervention often facilitated the adoption of a new understanding of pain that could reduce fears and worries in the long term. For instance, P05 mentioned that active listening was important to reduce worries and fears about back pain and previous experiences of not being understood: *“And have that reassurance that they're listening to when you have concerns and fears… relationship is really important.”* P05. However, for some participants, the information provided was experienced as confronting, conflicting with deeply held beliefs that pain is a sign of tissue damage. For some, the pain education implied that their pain was “not real” and “all in their head.” When describing this, it was common for participants to use the third person (ie, to explain how they perceived others would feel receiving the information). P06, for example, expressed that the PE narrative could be misinterpreted or misunderstood, resulting in people feeling that their pain experience was “invalid”: *“A lot of people would struggle to get their head around the idea, especially because maybe on the surface it's almost like what you're saying to a person is, you know, your pain is in your head, and they would misinterpret that to mean that your pain is not real.”*

After the interviewer reassured them that there were no wrong or correct answers, a few participants admitted to similar experiences at some point in the program. P11 highlighted the importance of the learning process to “get over the perception” that pain was not real: *“I think that there is a distinct proportion of people who would be quite sceptical about being told something that they interpret as this problem that I feel is all in my mind. It takes some understanding to get over that perception that that's what you're being told… what's underlying the problem.”* Conversely, P09 reported that the concept of PE was decisive in weakening the “pain was not real” idea: *“I soon started to learn that it was about the brain, it was about the neuroplasticity of the brain, and I had a maladaptive understanding of chronic pain, and my brain was alarmist in a lot of different ways.”*

For 2 participants who also had poor recovery (Table 1), the intervention was not enough for them to change the notion that “pain was not real.” P07, for example, denied “being a crazy person” after receiving the intervention: “*I cannot just have created this in my head because I think you would literally be a crazy person if you did that.”* P07.

### 3.2. You don't understand; my pain is different

All participants incorporated an understanding of the brain and central nervous system and considered the mind–body connection to make sense of their current pain experience. P07, for example, reported that the explanation of how pain works should be delivered early in their journey: *“I learned a lot, and it changed the way I think about pain and how it works. It was very interesting for someone who is been in pain on and off for my entire adulthood…. I think manipulating the brain to help manage how we feel or experience is essential*.” This cognitive process was related to less fear and worry but rarely excluded the possibility of structural issues on their backs, as described by P06: “*I have very clearly got an injury on my disc… you know, at a quite young age, and my pain now gets better and worse, and I'm very aware of the mind-body connection with that, but I'm not that worried about it anymore*.”

Some participants reported reasons why fear and worries about spinal problems persisted even after a comprehensive PE-grounded intervention. For example, P07 reported pain for a long time: “*…there is clearly something wrong with my back. I cannot be in this much pain, and maybe nothing wrong.”* P18 reported strong beliefs about abnormal spinal findings in his back: “*The MRI showed that my vertebrae are misaligned… Unfortunately, the pain is too severe for it to go away…”*

P16 perceived that the concept of an overprotective nervous system was invalid in her case because, having participated in the PE-grounded intervention, she no longer had movement apprehension or exaggerated appraisal of threat during functional tasks but still experienced pain: “*I'm not sure that the training I did can benefit the sort of pain I've got because it [the pain] occurs when I do certain things, and I'm not expecting the pain.*” Finally, P19 who had sought other physical interventions since participating in the PE-grounded intervention and attributed these interventions to improvements in pain intensity perceived this was “proof” that their pain persistence could be attributed to “something physical”: “*I still believe there's something physical there, for the simple reason that there are a specific set of things I can do to get relief, instant relief* (eg, “*foam roll my quads, release my hamstrings, get a trigger point, stand up and be in a lot less pain”*). For these 2 participants, PE-grounded interventions did not make sense because their symptoms did not match their understanding of the program's explanation and focus.

### 3.3. I'm unsure how to fit it into my daily life

Participants reported that experiential components, including physical activities, exercises, movement, and psychological explorations, help put theory into practice. Many participants found a way to apply education concepts to their lives and continued to do so one or 2 years after being exposed to the program. P10, for example, expressed that combining practical aspects with PE concepts increased the sense of agency and reduced worries about the consequences of having back pain: *“I just think that being active is the best thing I can do for my back*; *it just keeps the muscles and my body active, and my blood flow healthy. It also allows my brain to let go of those sensitive spots. It allows my brain to overcome those sensitive areas. So, by exercising, my brain receptors are just letting go of that protective mechanism*.”

However, some participants reported difficulties applying PE concepts in their daily routines. P16 was worried about not being able to deal with a possible experience of pain in the future: *“I'm not sure I learnt anything that I could put into practice. … there was nothing that said in future, when you experience this pain, this is what you should do.”* Similarly, P17 appeared to understand PE concepts but lacked self-efficacy in applying the knowledge in daily life, which probably would have reduced fears and worries of future pain experiences: *“It's all about your brain and your neurons and the gateways. I don't know how you apply that. I couldn't tell you exactly [what I did] because it's long ago. There have been many things happening in my life… once it [treatment] stopped, I think I forgot everything.”* These participants lacked a concrete activity where the concepts and experiences of the PE-grounded intervention could be applied in their daily lives, particularly in reducing pain or regulating their emotions. P20, among other participants, suggested that “refreshers,” additional psychological skills, or tools to practice the learning during pain situations would be helpful to reinforce knowledge learned in PE-grounded interventions: “*I think I probably need an app in my head or something like that to hear every day, that just tells me to do certain things to get my mind away from the actual pain that I think I'm feeling in my lower back.”*

Most participants reported that accepting the theory and applying their learning in practice required reinforcements from multiple actors in the health landscape (eg, family, media, the public, patients, and health professionals). P08, who did not recover from back pain, implied that having a community would help him deal with the structure-oriented messages about back pain: *“There were very, very sceptical views from anyone that I explained it to. It wasn't like there was a massive support network out there saying wow, that sounds like a great idea.”* By contrast, P05, who recovered from back pain, mentioned her effort to explain the ideas to friends and family members: “*…when you start talking to people… and explain [program, theories] to them, a lot of people could not understand it, and they asked me questions, and they challenged me, and there were things that I couldn't answer.”*

## 4. Discussion

This qualitative study explored people's beliefs and feelings about back pain 12 months after participating in a PE-grounded multimodal intervention, focusing on why fears and worries might persist. We identified 3 themes: “Are you implying my pain is not real?”; “You don't understand; my pain is different,” and “I am unsure how to fit it into my life.”

### 4.1. Are you implying my pain is not real?

The findings suggest that aspects of the intervention or its delivery may create the perception (usually transitory) that the clinician did not consider participants' pain experiences to be real. This misunderstanding diminishes as rapport between participant and clinician improves during the treatment program, and participants better understand PE concepts over time. However, a few people in our study perceived that the PE-grounded intervention implied they were at fault for their pain experience and reported feelings of invalidation. These same participants also experienced limited change in pain intensity and perceived recovery. This is consistent with findings from qualitative research in CLBP, which found that feelings of invalidation are associated with increased distress and disengagement with healthcare.^7,11^ Prioritising the relational aspects of back pain care (eg, listening to the pain experience, acknowledging fears and worries, validating responses, and providing cognitive and emotional reassurance) is essential for clinicians to establish trust with people seeking care and create a nonthreatening context in which to offer new information and form new understandings of pain.^24^ When people with back pain feel validated, this provides a foundation from which to challenge any unhelpful beliefs about back pain that commonly imply vulnerability in the back.

### 4.2. You don't understand; my pain is different

Beliefs about back pain based on a biomedical understanding are widespread and predate the onset of pain.^12^ During the PE-grounded intervention, people appear to add to their “repertoire of understanding” the belief that persistent pain may result from an overprotective nervous system. However, they seem to rarely exclude the possibility of having structural or biomechanical reasons for their pain experience. This finding is consistent with a dynamic adaptation process that involves assimilation and accommodation of a new experience.^17,19^ Our results indicate that some participants are assimilating new information by fitting it into their existing schema for the persistence of pain, but they still need to shape the pain schema with experimentation. The accommodation phase appears to be more challenging for people with CLBP who have experienced pain for a long time with great intensity, those who experience limited improvement in pain intensity during the intervention, and those who experience a disconnect between concepts of PE and the lived experience of bodily symptoms (eg, physical self-management approaches that subsequently relieve pain intensity and reinforce structural influences in the pain experience).^29^

Substantial effort has been made to develop treatments combining experimentation approaches and pain education.^1,9^ These treatments can monitor “the extent to which the participants understand the targets of the intervention and how it works”^32^ to capture incoherence between what has been told in the education and what people have been invited to do in the practical component—inconsistency between these components has been suggested to interfere with the recovery and lead to the persistence of fears and worries in some people.^28,31^ For example, self-management strategies strongly relying on “physical” approaches (eg, releasing the muscles with a rubber ball) might reinforce the belief that pain is due to structural or biomechanical factors and, at the same time, reinforce fears and worries that something serious is happening in the spine. To change deeply held beliefs about the vulnerability of the back, clinicians should consider monitoring intervention coherence, using pain communication skills (including PE), adding congruent exposure approaches to build confidence, and gradually progressing tasks toward the patient's needs and goals.^9,37^ In addition, clinicians could help people understand the reasons for choosing one strategy among others, guide them to consider strategies that bring long-term benefits,^24,33^ and equip them to focus on long-term benefits instead of strategies with immediate effects that may reinforce the idea that the body is in danger.

### 4.3. I am unsure how to fit it into my life

The challenges of integrating the learnings of pain education into participants' routines may explain why the benefits of PE, integrated or not with other approaches, often fade after 3 months.^38^ Additional strategies such as booster sessions (face-to-face or virtual), audio recording, virtual reality, and mobile apps containing information consistent with PE content might extend the intervention's benefits.

Although the biopsychosocial model has been encouraged for almost 5 decades, the dominant biomedical way of thinking may continue to be reinforced by family members, friends, social media, and other health professionals throughout PE.^2,25^ Some patients who described social challenges (eg, minimisation by others of the ideas proposed in PE had poor recovery) (eg, P08). By contrast, others had a good recovery (eg, P05). The opportunity and participants' effort to share the program's rationale and activities, as well as psychological regulation to deal with potential disagreements from the participants' view, appeared to influence the impact of interpersonal relationships on people's experiences. Some actions have been made in this direction, including appropriate pain curricula for healthcare providers, dissemination of PE to the public using social media and forums, regional/global initiatives such as Pain Revolution in Australia and Flippin' Pain in the United Kingdom, and the inclusion of family members in PE sessions.^1^

### 4.4. Clinical implication

Changing beliefs in the clinical setting is not linear; it depends on how patients come to the session (eg, mood, beliefs, skills) and how clinicians and society interact with patients during and after the clinical environment, respectively.^19^ In the end, it seems that the quality of the pain communication is determined by patients' interpretation and feelings during the interaction and how it will influence their health decisions. In Table 2, we provide suggestions on identifying, monitoring, and addressing emotional and cognitive responses that emerge during clinician–patient and society–patient interactions.

**Table 2:** Recommended questions and interaction during an individualised pain education–grounded intervention.

General questions to check possible maladaptive cognitive and emotional responses
*Before asking questions, consider using verbal and nonverbal validation cues, including active listening, such as “I understand what you feel”* or *“It sounds like…” [repeat what the patient has just said to you to show that you noticed his/her thoughts and feelings]*
What is your understanding of what you have learned so far?
How does what you have learned make you feel?
How does what you have learnt apply to your own condition and pain experience?

Validation of people's experience before a question or after people's responses
*Before replying, consider using verbal and nonverbal validation cues, including active listening, such as “I understand what you feel” or “It sounds like…” [repeat what the patient has just said to you to show that you noticed his/her thoughts and feelings]*

Integrating questions for checking cognitive and emotional responses with validation and educational approaches		
Maladaptive response	Compelling questions to expose the cognitive and emotional responses	Suggestions of actions
“Are you implying my pain is not real?” Misunderstanding that pain is consciously or unconsciously created by the person	[initial part of the program]. Can you explain to me why your pain is persisting or what you have heard about your pain? [during the program]. The way we understand pain may change depending on the knowledge, experience, previous information, and reflections about the topic. Can you explain to me why your pain is persisting? What has changed since you spent time reflecting on this topic with me?	Validate patients' points of view. Highlight the bioplasticity of the pain system (biological aspects that explain the overprotection and the possibility of change of these biological aspects for pain relief). Comment on the history of pain science and acknowledge that our current scientific understanding of the pain experience has evolved and this has not yet spread to the broader public. Validate patients' points of view. Include information (using different strategies such as different analogies, stories, facts, and experiential learning) to enrich the patient's repertoire or consider alternative points of view that may create empowerment.
“You don't understand; my pain is different” Persistent belief that the back is vulnerable and in danger	You have had pain for [pain duration]. You are an expert in your condition, and you may have heard many things about your pain. Today, why do you think your pain is persisting? What reason comes to mind when you have a flare-up? In your view, what are the contributors to your pain? What kind of treatment do you think you need to treat your pain? How do you think [the intervention patient is receiving] would benefit your condition?	Validate patients' points of view. Tailor the educational content to their specific challenging situations, such as longer pain duration and greater pain intensity; experiencing inconsistency between the pain education content and patients' experiences; attributing changes in pain intensity immediately after self-management; not experiencing satisfactory pain reductions with the treatment; receiving contradicting messages from different sources. Include information (using different strategies such as different analogies, stories, facts, and experiential learning) to enrich the patient's repertoire or consider alternative points of view that may create empowerment. Frame evidence-based interventions to be consistent with pain education content to treat chronic low back pain. Reinforce patients' ideas related to cognitive, emotional, and behavioural responses consistent with recoveries such as active behaviours, problem solving, and self-regulation.
“I'm unsure how to fit it into my life.” Uncertainty about how to make sense and apply the knowledge and experience to daily life	How do you see you applying these concepts into your daily life? What do you need to be more confident at applying the concepts to your daily life? What are the possible barriers that would interfere with your practice? What else do you need? What would be the tiniest sign of improvement? What is the smallest indicator that suggests your pain system is returning to a normal state? What change would you need to see to be reassured things are improving? What do you need from others or from the environment you live in to help you apply the concepts to your life?	Include accessible and preferential materials to reinforce pain educational contents such as booster sessions, experiential activities at home, audio recording suggestions, virtual reality, and mobile app activities. Remind patients about bioplasticity and that the changes in the pain system occur without their awareness; therefore, it may take some time to perceive improvements. It may encourage patients to apply the knowledge in their daily life. For patients: Include family members in some pain education sessions, develop forums where patients can inspire others with pain education concepts; prepare patients to deal with opposing viewpoints (eg, leaflet to give people about the main idea of pain education). For health care providers: An appropriate pain curriculum that includes pain education and communication. For the public: mass pain education campaigns.

### 4.5. Design considerations and limitations

A potential limitation is the absence of people with lived experience of CLBP from the research team and data analysis. Another key limitation of the study design is that interviews were conducted at a single time, one or more years after the intervention; therefore, we relied on memory recall. However, this study aimed to investigate the persistence of fears and worries over time; thus, the timing of the interviews was deemed appropriate. Twenty interviews were sufficient to capture our target for diversity in experiences and identify response patterns to answer the research questions. Findings from this sample may not be transferrable to people with CLBP living in developed countries and seeking treatments in primary care. Future research is required to investigate the experiences of people from diverse cultural and linguistic backgrounds participating in PE. Pain education is constantly evolving, and the findings of the qualitative study cannot be generalised to all types of PE-grounded interventions.

## 5. Conclusion

The results of this qualitative study provide a basis for monitoring and managing critical emotional and cognitive responses that emerge during PE-grounded multimodal interventions that might interfere with fear and worry management in people with CLBP.

## Disclosures

R.R.N.R. received fees from the 2021 Allied Health Cross-Boundary Grant Stream to facilitate a workshop about pain education and clinical hypnosis. G.L.M. has received support from Reality Health, ConnectHealth UK, Kaiser Permanente, AIA Australia, Workers' Compensation Boards, and professional sporting organisations in Australia, Europe, South, and North America. Professional and scientific bodies have reimbursed him for travel costs related to research presentations on pain and pain education at scientific conferences/symposia. He has received speaker fees for lectures on pain, pain education, and rehabilitation. He receives royalties for pain and pain education books, including 3 books used in, or directly relevant to, the RESOLVE intervention. M.K.B. has received fees to speak about pain neuroscience and rehabilitation and engagement with research evidence. The other authors declare that there is no conflict of interest.

## Supplemental digital content

Supplemental digital content associated with this article can be found online at http://links.lww.com/PR9/A253.

## Supplementary Material

SUPPLEMENTARY MATERIAL
