# Supplementary material for: Why might fears and worries persist after a pain education–grounded multimodal intervention for chronic back pain? A qualitative study
Source: Pain Rep. 2024 Nov 13;9(6):e1197. doi: 10.1097/PR9.0000000000001197 (PMC11563001; doi:10.1097/PR9.0000000000001197)
Supplement: SUPPLEMENTARY MATERIAL [file painreports-9-e1197-s001.pdf]

Supplementary material 1: Interview guide

Warming up:

- Please tell me about your current experience with back pain.
- How long have you had low back pain? Or how long had you had low back pain?
- Can you talk me through your health-care journey?
- What do you think caused your pain?
- Why do you think your pain is persisting?
- What kind of treatment do you think that would be good for you?

[Interviewer summarizes the statements, here]

Questions [the order of the questions depended on the conversation flow]:

Perception about interventions that target the brain for CLBP [the analyst focused on people's experiences in the pain science education treatment component].

- What role does the brain play in your pain?
- What do you think about treatments for pain intended to target the brain instead of targeting only the structure of the back?
- What are the reasons that you see to target the brain when someone has pain for long time?
- What kind of treatment comes to your mind when you hear about treatments trying to target the brain to treat back pain?
- What kind of those treatments do you think that would be good for you? Why?
- How do you think people will see treatments intended to target the brain for treating low back pain?

Experience in the pain education-grounded intervention:
Now, I will ask you specific questions about your experience in the treatment program you received as part of the clinical trial [the analyst focused on people's experiences in the pain education treatment component].

1. What are your overall thoughts about the treatment you received?

2. How did the treatment you received compare with previous treatments, or other treatments you were receiving at the time?
3. How was your experience in putting into practice what you learned during the programme? How did you apply your experience during the session into your daily life?
4. What did you find easiest to comply with? Why?
5. What did you find hardest to comply with? Why?
6. What do you think is missing from the treatment?
7. How did you explain the purpose/aim of the programme to your loved ones or friends?
8. How did you make sense of this approach in relation to your back pain? (look for how they matched their cause in the preliminary questions to what we tried to change)
9. How confident did you feel about your skills to move forward on your own at the end of the intervention?
10. What about after the trial, would you have liked to be able to talk to the clinician again? Why?

Supplemental material 2. Themes, codes and quotes demonstrating why fears and worries might persist with pain education-grounded intervention.

**Theme: "Are you implying my pain is not real?"** People's belief that pain education may imply that pain is consciously or unconsciously created by the person	
**Specific code in the theme**	**Qualitative excerpts**
Establishing sensitive and strategic clinician-patient communication	*I think that it will need to be something that's approached carefully and sensitively with some patients.* P11
Creating negative feelings of blame	*I cannot just have created this in my head because I think you would literally be crazy person if you did that.* P07
Creating negative feelings of punishment	*I don't think subconsciously I want to punish myself and remain in pain. I do not understand the logic behind what was explained.* P08
**Theme: "You don't understand; my pain is different"** People's persistent belief that structure problems are responsible for their pain experience.	
**Specific code in the theme**	**Qualitative excerpts**
No convincing differentiation between structural causes of pain and brain influences	*…a posture assessment, you know, I thought that would be good. Other than that, maybe every person, they sort of have their back x-rayed, in some sort of way, and then that way people who still have a belief that it's something wrong with their spine can actually be shown that their spine is okay; it's genuinely.* P20
Duration and severity of pain	*Still not 100% convinced there is not something wrong with my back because I truly do not understand how you can experience pain for years and years and years, and there is not something wrong. … there is clearly something wrong with my back. I cannot be in this much pain and maybe nothing wrong".* P07
Incomplete relief with interventions based on pain education content	*The MRI showed that my vertebrae are misaligned… Unfortunately, the pain is too severe for it to go away…* P18
Pain education concepts not consistent with people`s own experiences	*I'm not sure that the training I did can benefit the sort of pain I've got, because the pain I've got occurs when I do certain things and I'm not expecting the pain.* P16
Subsequent and regular relief with physical self-management approaches	*I still believe there's something physical there, for the simple reason that there are a specific set of things I can do to get relief, instant relief.* (e.g., *"foam roll my quads, release my hamstrings, get a trigger point, stand up and be in a lot less pain"*) P19
**Theme: "I am unsure how to fit it into my life"** Lack of confidence in integrating the knowledge to recovery or manage pain	
**Specific code in the theme**	**Qualitative excerpts**
Physical component as a critical element for learning	*I am a concrete learner, so I need to have that physicality so, … if we did not have any physical activity attached to the talking about it would not have worked for me. I needed to be able to go away and have something to do.* P02
Absence of adjunct behavioral approaches	*It is probably end the brain, not just… I do not think you can just have someone and feeling poor physical health or overweight or is got whatever going on… you cannot just target the brain.* P06
Absence of exercise or physical activity approaches in the pain education intervention	*I think it would be good to get people out to actually get physical. Someone who is reading the stuff may not necessarily go out and do it, not necessarily go out and exercise.* P10
Combining *meaningful* physical activities to complement the pain education	*He [researcher] told me to do these little exercises and stuff. I don't think I did them, but I go to my gym, and I do other exercises and it's back-strengthening exercises, so I do other stuff.* P17
Combining psychological approaches to reinforce,	*Before the trial, I was trying to relax the back [using hypnosis] and I did get some good results, so when I was at the study it helped me because I already could see a*

consolidate or complement the pain education	*bit of the relationship between the mind and the body. And to me it helped and during the study, a few things came back of how mind things work.* P03
Concepts of pain education dissipate over time	*I think life gets in the way people get busy and I forget. But it certainly it is all stuck in my head, and I am very conscious of it and I still talk about it to people, but I think when you get very busy, it is easy to forget.* P09
Missing reinforcement of the pain education concepts	*I think I probably need an app in my head or something like that, some sort of – to hear every day, that just tells me to do certain things to get my mind away from the actual pain that I think I'm feeling in my lower back.* P20
Missing practical components to deal with flare-ups	*I'm not sure I learnt anything that I could put into practice. … there was nothing that said in future, when you experience this pain, this is what you should do.* P16
Previous personal experiences	*I can only think after a millennium of personal experiences that whatever you bang, or hit is the thing that's sore because you've banged or hit it.* P08
Lack of brain-oriented treatments in society	*So, initially I would think people would scoff but the wide community is being more exposed by being made aware of these mind-altering drugs* P02
Excessive reinforcements of biomedical view from health professionals (imaging)	*When you go to a physio and the physio do not want to touch you unless she is got a picture of what she is looking at… but that is the way the medical world has created us. The way we have been made to think that you need to see something before you work on it.* P02
Culture of biomedicine	*I think the biggest issue for people is that not being heard, and if they have a cultural belief system of medication or a tablet will fix it, or an operation will fix it, then that makes it even harder.* P09
Lack of social network to encourage the use of pain education	*There was very, very skeptical views from anyone that I explained it to. It wasn't like there was a massive support network out there saying wow, that sounds like a great idea. Everyone just said that's absolute rubbish.* P08
Lack of successful examples in the use of pain education	*I'd be curious about real life examples of people who solved their back problems through applying these theories, these practices. I would love - I've never heard… because they must know something that I don't, and again I want to get the magic ingredient, what is that I'm not doing, I'm missing.* P19
Age, education background and severity of the condition	*… for me was fine but how it would apply for someone from a diverse social cultural background or someone that has a clear a disability or people with lower levels of literacy, you know, like if the intervention actually works*. P06
